# proGenomes2: an improved database for accurate and consistent habitat, taxonomic and functional annotations of prokaryotic genomes

**DOI:** 10.1093/nar/gkz1002

**Published:** 2019-10-24

**Authors:** Daniel R Mende, Ivica Letunic, Oleksandr M Maistrenko, Thomas S B Schmidt, Alessio Milanese, Lucas Paoli, Ana Hernández-Plaza, Askarbek N Orakov, Sofia K Forslund, Shinichi Sunagawa, Georg Zeller, Jaime Huerta-Cepas, Luis Pedro Coelho, Peer Bork

**Affiliations:** 1 Department of Medical Microbiology, Academic Medical Centre, University of Amsterdam, Amsterdam, The Netherlands; 2 Biobyte solutions GmbH, Bothestr, 142, 69117 Heidelberg, Germany; 3 Structural and Computational Biology Unit, European Molecular Biology Laboratory, 69117 Heidelberg, Germany; 4 Institute of Microbiology, Department of Biology, ETH Zurich, Vladimir-Prelog-Weg 4, 8093 Zurich, Switzerland; 5 Centro de Biotecnología y Genómica de Plantas, Universidad Politécnica de Madrid (UPM) - Instituto Nacional de Investigación y Tecnología Agraria y Alimentaria (INIA), Campus de Montegancedo-UPM, 28223, Pozuelo de Alarcón, Madrid, Spain; 6 Max Delbrück Centre for Molecular Medicine, 13125 Berlin, Germany; 7 Institute of Science and Technology for Brain-Inspired Intelligence, Fudan University, Shanghai, China; 8 Key Laboratory of Computational Neuroscience and Brain-Inspired Intelligence (Fudan University), Ministry of Education, China; 9 Molecular Medicine Partnership Unit, University of Heidelberg and European Molecular Biology Laboratory, 69120 Heidelberg, Germany; 10 Department of Bioinformatics, Biocenter, University of Würzburg, 97074 Würzburg, Germany

## Abstract

Microbiology depends on the availability of annotated microbial genomes for many applications. Comparative genomics approaches have been a major advance, but consistent and accurate annotations of genomes can be hard to obtain. In addition, newer concepts such as the pan-genome concept are still being implemented to help answer biological questions. Hence, we present proGenomes2, which provides 87 920 high-quality genomes in a user-friendly and interactive manner. Genome sequences and annotations can be retrieved individually or by taxonomic clade. Every genome in the database has been assigned to a species cluster and most genomes could be accurately assigned to one or multiple habitats. In addition, general functional annotations and specific annotations of antibiotic resistance genes and single nucleotide variants are provided. In short, proGenomes2 provides threefold more genomes, enhanced habitat annotations, updated taxonomic and functional annotation and improved linkage to the NCBI BioSample database. The database is available at http://progenomes.embl.de/.

## INTRODUCTION

Large-scale genomics has been instrumental for our improved understanding of microbes. Microbiology has developed into a data-intensive field with the availability of thousands of sequenced genomes (1–3). Over the last 20+ years, the number of bacteria and archaea with sequenced genomes has grown exponentially (4,5). To facilitate an understanding of microbes from their genomic data, annotations are essential. These enable researchers to pinpoint potential functions and allow for comparative analyses (6). For this reason, we initially developed proGenomes and are continuing to improve the database. Several publicly accessible databases provide genomes with basic or even more elaborate annotations. For example, the NCBI RefSeq database (7) make a comprehensive set of genomes available to the public (though only minimal annotations are provided). Further, databases such as Ensembl Bacteria (8), the DOE’s Joint Genome Institute Integrated Microbial Genomes & Microbiomes (JGI IMG/M) database (9), or the PATRIC (Pathosystems Resource Integration Center) database (10) contain more sophisticated, but often select information and annotations. For these databases, the taxonomic annotations are selected by the submitter of each genome. This leads to inconsistencies across different clades across the tree of life, especially at the species level, as the species definition for bacteria and archaea remains a highly debated topic among microbiologists (11,12). In general and not only due to user errors, inconsistencies are wide-spread in genomic databases (13–15). A successful effort to increase the consistency of the taxonomy at higher taxonomic levels is the Genome Taxonomy Database (GTDB) (12), while specI (5) using genomics information to delineate species was used in proGenomes v1 (4).

The pan-genome concept has been an important advance in microbial genomics and microbiology overall (16,17). Due to the availability of many genome sequences within one species, researchers can now explore the pan-genome of many species and study the functional repertoire of species. Still most genomes are studied on an individual basis even in comparative approaches. Dedicated databases for pan-genomes exist, but these are often focused on specific taxonomic clades or lack in-depth functional annotations. Hence, the availability of pan-genomes for many species could facilitate many studies and applications

Here, we present proGenomes2 which was developed to address these issues as an update of the existing proGenomes database. The updated version provides three times as many genome sequences and annotations and a higher phylogenetic coverage while adding information about the pan-genome of every species cluster. A number of workflows were improved for proGenomes2 including enhanced habitat annotations and linkage to the NCBI BioSample database. The database is available at http://progenomes.embl.de/

## DATABASE CONSTRUCTION AND CHARACTERISTICS

proGenomes2 provides the available microbial genomes and customizable subsets in a readily downloadable and user-friendly manner. Genomes and sets of genomes can be found and retrieved using the taxonomic name of the organism, species or clade. The provided information can be accessed, explored interactively and downloaded easily. The database will be updated regularly in the future and major upgrades of the underlying computational pipeline are planned every two years. The genomes for proGenomes2 were obtained on 15 May 2017 and the NCBI taxonomy database used was downloaded on 8 January 2019.

### Genome collection

We downloaded all bacterial and archaeal genomes that were available from the NCBI Nucleotide database on 15 May 2017. Gene annotations provided with the deposited genomes were used when available. If gene annotations were absent from the deposited genomes, we used geneMarkS to predict genes (18). To exclude low quality genomes, we used the same parameters as used in the original proGenomes version (N50 score >10k bp, <300 contigs and >30 of 40 universal, single copy marker genes (19,20)). We further removed genomes that were since removed from the NCBI Nucleotide database and genomes that we found to be of low quality by manual quality filtering. After these filtering steps, 87 920 high-quality genomes remained (10 333 genomes were removed). In comparison, proGenomes version 1 contained 25 038 high-quality genomes. proGenomes2 normalizes genome, gene and protein identifiers to a consistent scheme, linking them to NCBI taxonomic and BioSample IDs, to facilitate downstream automated processing. This also ensure access to information about the sequenced sample that is provided by the NCBI BioSample Database (21)

### Species clusters definitions using the specI approach

specI species clusters provide an accurate and consistent solution for genomics-based species definition that are largely consistent with consensus from morphological and phenotypic evaluation). We calculated specI species clusters as in the previous proGenomes version using the methodology described in (5), resulted in 12 221 specI species clusters for the 87 920 genomes currently in proGenomes2 (proGenomes version 1: 5306 specI cluster). In short, pairwise genome-to-genome identities were calculated as a length-weighted average of the nucleotide identities of a set of 40 universal, single copy marker genes (19,20) calculated with vsearch (v1.8.0) (22). These were transformed into distances and clustered using average linkage employing a cutoff of 3.5% distance (96.5% nucleotide ID). Genomes are annotated according to the NCBI taxonomy (downloaded on 8 January 2019 and available at https://doi.org/10.5281/zenodo.3357977). Annotation for the specI is derived from the annotation of the genomes that compose the specI clusters.

### Selection of representative genomes

Many species and even strains have been sequenced multiple times leading to an increasing amount of redundancy in genomic databases. Non-redundant datasets are increasingly important in microbial genomics (23). Hence, proGenomes provides a non-redundant set of 12 221 representative genomes and habitat-specific subsets. These datasets are precompiled and can be readily downloaded.

For each specI cluster one representative genome was selected. For this purpose, a whitelist containing several highly-important genomes was compiled. For all specI clusters that contained a genome on the whitelist, this genome was selected as representative. For all other specI clusters, the representative genomes were selected using citation counts as well as the N50 measure as a proxy for genome quality, while completely assembled genomes were selected preferentially.

### Pan-genomes

**Figure 1.**
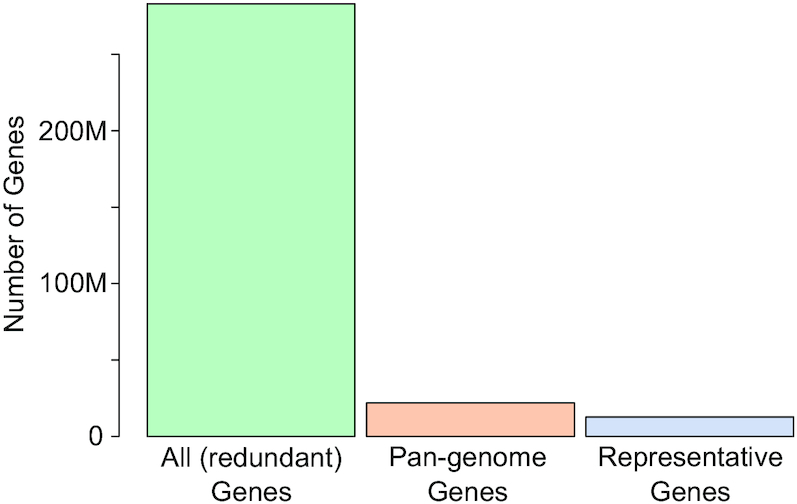
Cumulative number of genes in specI clusters with more than one genome. Number of all redundant genes (left), genes represented in pan-genomes (middle) and genes from representative genomes (right).

In addition to representative genomes, proGenomes provides pan-genomes for the specI clusters. These are non-redundant sets of genes that represent the genetic diversity within a specI (species) cluster. Per specI-cluster pan-genomes were generated in two steps. First, genes were de-replicated by sorting the genes and removing identical sequences. Second, these dereplicated gene sets were further clustered with cd-hit-est (24) to produce non-redundant versions, at 95% identity and 90% coverage (exact command used: -c 0.95 -G 0 -g 1 -aS 0.9) (25). The resulting pan-genomes can be used for many applications ranging from metagenomic mapping to evolutionary analyses of functional genes across different species. For clusters with more than one genome, this reduced the number of genes from 283 million to 63 million, while providing a far greater coverage of the functional repertoire as the representative genomes alone (21.8 million) (Figure 1).

### Functional annotation

The functional repertoire of a microbial genome defines its phenotype, lifestyle and ecological role. Hence, it is pivotal to our understanding of a microorganism that we have a consistent, accurate and comprehensive functional annotation of its genes. One of the main aspects of proGenomes is to provide consistently-computed functional annotation of protein-coding genes. For general annotations, we use the eggNOG-mapper for eggNOG 5.0 (26) software that assigns protein-coding genes to orthologous groups which are in turn assigned to functions and broader functional categories.

proGenomes2 provides dedicated antibiotic resistance annotations of both antimicrobial resistance genes and resistance-conveying single nucleotide variants. The antibiotic resistance annotations in proGenomes2 are provided based on integrated results from the Comprehensive Antibiotic Resistance Database (23,27) and ResFams (28) resources as in proGenomes version 1. Since both databases (CARD v3.0.0 and ResFams v1.2.2) map to the antibiotic resistance ontology (ARO), the ARO hierarchy (as per CARD version 3.0) was used to assess which antibiotics each resistance gene determinant protects against. Proxy terms for ‘unspecified beta-lactam’ and ‘multidrug efflux pump’ were added to reconcile ambiguities in some annotations. For complexes listed in the ARO, such as components with disparate subunits, such synergies between hits were counted within each genome, reflecting how the presence of several interacting antibiotic resistance genes can provide further resistance. Overall, almost 288 million protein-coding genes were annotated using eggNOG (proGenomes version 1: 80 million). This information can be explored interactively on the proGenomes website.

### Habitat information

**Figure 2.**
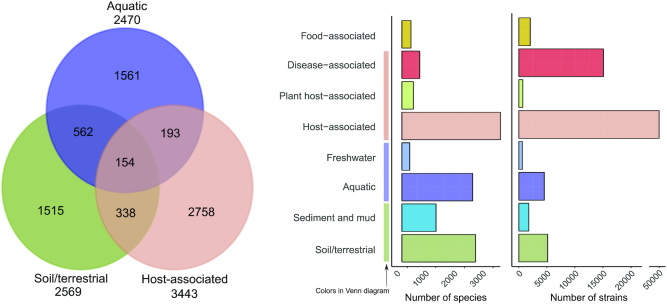
Habitat annotations. In proGenomes2 organisms can be associated with multiple habitats categories. The left panel shows the overlap between specI species cluster habitat annotations. The right panel shows how many species (clusters) and strains are associated with each habitat category.

proGenomes2 provides annotations of each genome and species to a habitat. As previously, the habitat annotations are based on the PATRIC database. Information regarding the isolation source was parsed from Patric database version 3.5.28 (accessed on 8 December 2018) (29). Habitat annotations are available for 7218 out of the 12 221 specI clusters (59 344/87 920 isolates). Species were considered associated with a habitat if at least one genome was isolated from that habitat. We expanded the habitat classification from the previous version and provide three broad (soil-associated, aquatic, host-associated) and additionally five more specific categories (mud/sediment, freshwater, disease-associated, food-associated)—biologically meaningful categories (Figure 2). Representative genomes for these subsets of clusters are available for bulk download from the website. We removed the category ‘multiple’ used in the previous version and allow the annotation to more than one category in proGenomes2. As many genomes were annotated as food-associated, isolated from sediment or freshwater, we introduced these as a new categories. Annotations are provided with each genome and specI cluster as well as one large downloadable file.

### Website

To navigate and access the proGenomes2 database, we provide a website (http://progenomes.embl.de). A search function allows users to access data for specific taxonomic groups or specI clusters directly. Information about single genomes is provided interactively with direct links to relevant external database information, while for higher level taxonomic groups concise information about the organisms belonging to that group are displayed.

The website further allows users to provide their own genomes for annotation and contextualization within the proGenomes framework. Taxonomic placement with respect to the above-described specI clusters proceeds in four steps. First, genes and proteins in the uploaded genome are identified using Prodigal (30). Second, among these genes the 40 marker genes on which the specI clusters were defined are identified using the methodology described in (5). In a third step, the extracted marker genes are compared to those from the 12 221 specI clusters using vsearch (22) with the gene-specific thresholds introduced above. Finally, a consistent annotation for the genome is derived from the mapping of individual marker genes (more details are documented on the progenome website). This taxonomic placement tool is also available as stand-alone software. Functional annotations via the eggnog-mapper web server (31) are accessible via a link.

### Future outlook

We are planning to upgrade proGenomes further in the near future. The upcoming improvements include the integration of the GTDB taxonomy and the inclusion of metagenomics-assembled genomes (MAGs).To enable this we aim to improve our quality measures for genomes in general and with a specific focus on issues related to MAGs.

## DISCUSSION

proGenomes provides consistent taxonomic and functional annotations for quality filtered genomes, as well as a non-redundant, habitat-specific sets of representative genomes. The easy-to-use website provides a wide range of information relevant to researchers interested in microbial genomics and allows the customization of subsets of genomes for download, thus facilitating comparative studies that address questions from evolution, population genetics, functional genomics and many other research fields. We intend proGenomes to be a valuable resource for studies ranging from those focusing on one or a few organisms to those analyzing large-scale evolutionary patterns or complex microbial communities.
